# Markerless Liver Tumor Localization Using Internal Liver Volume Delineated By Four-Dimensional Cone-Beam CT

**DOI:** 10.7759/cureus.14465

**Published:** 2021-04-13

**Authors:** Naohisa Mizukami, Kiyoshi Yoda

**Affiliations:** 1 Department of Radiation Oncology, Yame General Hospital, Yame, JPN; 2 Research Physics, Elekta K.K., Tokyo, JPN

**Keywords:** markerless, liver tumor, localization, four-dimensional cone-beam computed tomography, internal liver volume, 4d cbct, lipiodol, image guided radiotherapy, igrt

## Abstract

Markerless liver tumor localization has been proposed using an internal liver volume delineated by four-dimensional cone-beam CT (4D CBCT). Liver CT was performed under mid-ventilation breath hold, and transferred to a treatment planning system (TPS) to contour the gross target volume (GTV). Subsequently, liver 4D CBCT was performed and transferred to the TPS. After bone matching between the CT and the 4D CBCT, an internal liver volume was delineated on the liver CT volume as the union of liver volumes within a breathing cycle of the 4D CBCT volumes. Then, inhale liver volume was delineated on the 4D CBCT. Next, the internal target volume was defined by expanding the GTV by referring to the liver movement within the respiratory cycle of the 4D CBCT. Subsequently, all the delineated structures were transferred to the 4D CBCT unit. Immediately before treatment, 4D CBCT was performed again and the couch was repositioned so that the liver may move superiorly to the internal liver volume boundary and inferiorly to the inhale liver volume boundary during the respiratory cycle. The target localization accuracy of the proposed method was evaluated by comparing it to a published lipiodol-based technique. Both methods were applied to a single case in which lipiodol remained inside the tumor. 3D couch repositioning vectors for the two procedures were collected for 25 fraction data of the above same patient, and the differences in the vectors were calculated. The target localization deviations of the proposed method in reference to the lipiodol-based procedure were 0.7 mm ± 0.9 mm (SD) in the lateral direction, 2.0 mm ± 0.7 mm (SD) in the superior-inferior direction, and -2.1 mm ± 0.8 mm (SD) in the anterior-posterior direction. Markerless liver tumor localization is feasible by delineating the internal liver volume and the inhale liver volume using 4D CBCT.

## Introduction

Lipiodol retention is regarded as a surrogate for cone-beam CT (CBCT) image guidance in radiotherapy of a liver tumor [1-3], allowing the tumor-to-tumor registration between planning and treatment stages. A limitation is that the lipiodol, which was injected at the time of interventional radiology, may be washed out from the tumor after a while, and it may be unavailable at the time of radiotherapy. This happens frequently to the liver tumor patients in our facility. In addition, lipiodol is available only when transcatheter arterial chemoembolization (TACE) was performed with the patient's consent. A metal marker can also be implanted for liver radiotherapy as an alternative technique. However, the invasive embedding of the marker may lead to serious complications such as bleeding. It is also known that the target localization accuracy decreases when the tumor-to-marker distance increases [4]. Recently, 4D CBCT has been widely employed for target localization for respiratory moving tumors [5]. The purpose of this study is to propose a markerless liver tumor localization using the moving liver volume as a surrogate which is delineated by 4D CBCT. The proposed technique may allow 4D CBCT image-guided radiotherapy for a liver tumor without using an external marker even when lipiodol is unavailable.

## Case presentation

A 73-year-old female patient was diagnosed with cirrhosis type C, and subsequently diagnosed with hepatocellular carcinoma (HCC) in segment six. TACE was performed twice, resulting in local control failure, and thereafter radiotherapy was ordered. Partial lipiodol retention was observed in the HCC.

Liver CT was performed under mid-ventilation breath hold, and transferred to a treatment planning system (TPS) to contour the gross target volume (GTV). Subsequently, liver 4D CBCT was performed and transferred to the TPS. After bone matching between the CT and the 4D CBCT, an internal liver volume was delineated on the liver CT volume as the union of liver volumes within a breathing cycle of the 4D CBCT volumes, where a concept of internal organ volume was first mentioned by Li et al [6]. Then, inhale liver volume was delineated on the 4D CBCT. Next, the internal target volume was defined by expanding the GTV by referring to the liver movement within the respiratory cycle of the 4D CBCT. Subsequently, all the defined structures were transferred to the 4D CBCT unit.

Immediately before treatment, 4D CBCT was performed again and the couch was repositioned so that the liver may move superiorly to the internal liver volume boundary and inferiorly to the inhale liver volume boundary during the respiratory cycle. To reduce the patient's respiratory amplitude, a vacuum-activated immobilization unit, BodyFIX (Elekta, Stockholm, Sweden) was employed.

The target localization accuracy of the proposed method was evaluated by comparing it to the lipiodol-based technique [1-3]. Both methods were applied to the present case in which lipiodol remained inside the tumor. 3D couch repositioning vectors for the two procedures were collected for 25-fraction CBCT data of the above patient, and the differences in the vectors were calculated.

Video 1 shows 4D CBCT images of a liver tumor on coronal (left) and sagittal (right) planes with intratumoral lipiodol retention. In this case, the contours of the tumor and the lipiodol are assumed identical. Immediately before treatment, the patient couch was repositioned so that the moving tumor with lipiodol stayed inside the internal target volume (ITV) in pink during a breathing cycle, where the ITV was defined in a TPS.

**Video 1:** 4D CBCT images of a liver tumor on coronal (left) and sagittal (right) planes with intratumoral lipiodol retention. 4D CBCT: Four-dimensional cone-beam computed tomography.

Video 2 demonstrates 4D CBCT images of a liver tumor on coronal (left) and sagittal (right) planes with a delineated internal liver volume (light pink) and an inhale liver volume (yellow). Immediately before treatment, the patient couch was repositioned so that the liver may move superiorly to the internal liver volume boundary and inferiorly to the inhale liver volume boundary during a respiratory cycle.

**Video 2:** 4D CBCT images of a liver tumor on coronal (left) and sagittal (right) planes with a delineated internal liver volume (light pink) and an inhale liver volume (yellow). 4D CBCT: Four-dimensional cone-beam computed tomography.

The target localization deviations of the proposed method in reference to the lipiodol-based procedure were 0.7 mm ± 0.9 mm in the lateral direction, 2.0 mm ± 0.7 mm in the superior-inferior direction, and -2.1 mm ± 0.8 mm in the anterior-posterior direction.

## Discussion

To the authors’ best knowledge, this is the first report proposing a markerless liver tumor localization using an internal liver volume and an inhale liver volume delineated by 4D CBCT. The major uncertainty of the proposed target localization was 2 mm in the superior-inferior and the anterior-posterior directions. Our liver treatment planning mostly employs an isotropic margin of 5 mm [7]. The above uncertainty is therefore clinically acceptable.

Because the means of the localization errors are significantly larger than the SDs in the above two directions, systematic errors are dominant factors. The systematic errors may result from our procedure of registering 4D CBCT and 3D planning CT using bone matching. By employing 4D planning CT, a more accurate registration may be performed. It was also reported that respiratory movement of the liver has rotational components [8], which may bring about some uncertainties in the proposed liver contour matching on the coronal and sagittal planes. However, the advantage of noninvasive liver radiotherapy outweighs the errors observed in this study.

In this case study, the tumor was located in the superior part. We have no data for a tumor in the inferior part, which may need another analysis.

Lastly, the proposed technique may be easily employed and verified in any institution having a linac with 4D CBCT functionality.

## Conclusions

We have proposed a new method for markerless liver tumor localization using an internal liver volume and an inhale liver volume delineated by 4D CBCT. Localization accuracy was measured in reference to the published lipiodol-based technique, leading to an uncertainty of 2 mm which was considered clinically acceptable.
